# Supplementary material for: Linkage between Psychological Factors and Response to Immune Checkpoint Inhibitor Therapy: A Preliminary Study
Source: Cells. 2023 Oct 17;12(20):2471. doi: 10.3390/cells12202471 (PMC10605722; doi:10.3390/cells12202471)
Supplement: Supplementary file 1 [file cells-12-02471-s001.zip › cells-2639147-supplementary.pdf]

Table S1. Differences in demographic variables between participants who participated or did not participate at T1.

	Participated at T1		Did not participate at T1		*t* or χ^2
	M or *n*	*SD* or %	*M* or *n*	*SD* or %	
Age (years, *M*, *SD*)	67.79	14.20	72.17	11.88	1.26
Sex (*n*, %)					0.13
Female	11	33.3	11	29.9	
Male	16	66.7	17	71.1	
Education (years, *M*, *SD*)	12.32	2.94	13.00	3.05	0.81
Marital status (*n*, %)					0.88
Married or partnered	25	65.8	17	70.8	
Divorced	8	21.1	4	16.7	
Widowed	4	10.5	3	12.5	
Single	1	2.6	0	0.0	
Income level[a] (*n*, %)					0.84
High	2	5.7	2	8.7	
Average	20	57.1	15	65.2	
Low	13	37.2	6	26.1	
Religion (*n*, %)					0.64
Jewish	31	81.6	20	83.3	
Arab (Muslim or Christian)	6	15.8	4	16.7	
Other	1	2.6	0	0.0	
Tumor type (*n*, %)					
Lung	13	34.2	5	20.8	
Melanoma	8	21.1	6	25.0	
Renal	5	13.2	3	12.5	
Breast	4	10.5	2	8.3	
Skin small-cell carcinoma	2	5.3	4	16.7	
Urinary bladder	2	5.3	2	8.3	
Colon	2	5.3	1	4.2	
Gastric	2	5.3	0	0.0	
Sarcoma	0	0.0	1	4.2	
ECOG PS at T0 (*n*, %)					3.47
0	14	36.8	14	58.3	

1	16	42.1	5	20.8	
2	8	21.1	5	20.8	
Type of ICI treatment (*n*, %)					0.65
CTL4+PD1[b]	12	31.6	10	41.7	
PD1/PDL1[c]	26	68.4	14	58.3	
Line of IT (*n*, %)					4.89
1	24	63.2	15	62.5	
2	6	15.8	8	33.3	
3	8	21.1	1	4.2	

[a] Calculated from actual responses.
[b] Ipilimumab and Nivolumab.
[c] Pembrolizumab, Nivolumab, Cimiplimab, Atezolizumab, Durvalumab.

Table S2. Associations among psychosocial variables.

	1	2	3	4	5	6	7
1. HRQoL (T0)							
2. HRQoL (T1)	.69***						
3. Perceived health (T0)	-.75***	-.53***					
4. Perceived health (T1)	-.73***	-.68***	.73***				
5. Emotional distress (T0)	.68***	.64***	-.65***	-.70***			
6. Emotional distress (T1)	.52***	.64***	-.41*	-.46**	.60***		
7. Sleep quality (T0)	.60***	.59***	-.56***	-.57***	.70***	.52***	
8. Sleep quality (T1)	.43**	.53***	-.52***	-.51***	.59***	.48**	.66***

*p < .05. **p < .01. ***p < .001.

Table S3. Associations among blood measures.

	1	2	3	4	5
1. TNFα					
2. IL-2	.19				
3.IL-6	**.47***	.28			
4. IL-10	-.08	.09	.07		
5. PD-1	-.11	.03	.09	.04	
6. CTLA-4	.-.01	.27	.10	-.03	**.50***

*p < .001.
